# Soluble markers of neutrophil, T-cell and monocyte activation are associated with disease severity and parasitemia in falciparum malaria

**DOI:** 10.1186/s12879-018-3593-8

**Published:** 2018-12-18

**Authors:** Kari Otterdal, Aase Berg, Annika E. Michelsen, Sam Patel, Marit G. Tellevik, Christel G. Haanshuus, Børre Fevang, Pål Aukrust, Nina Langeland, Thor Ueland

**Affiliations:** 1 Research Institute of Internal Medicine, Oslo University Hospital Rikshospitalet, PO Box 4950, Nydalen, 0424 Oslo, Norway; 2 Department of Medicine, Stavanger University Hospital, PO Box 8100, 4068 Stavanger, Norway; 3 Department of Medicine, Central Hospital of Maputo, 1100 Maputo, Mozambique; 4 Faculty of Medicine, University of Oslo, 0316 Oslo, Norway; 5 National Centre for Tropical Infectious Diseases, Department of Medicine, Haukeland University Hospital, 5021 Bergen, Norway; 6 Section of Clinical Immunology and Infectious Diseases, Oslo University Hospital Rikshospitalet, 0372 Oslo, Norway; 7 K.G. Jebsen Inflammatory Research Center, University of Oslo, 0424 Oslo, Norway; 8 Department of Clinical Science, University of Bergen, 5021 Bergen, Norway; 9 Department of Medicine, Haukeland University Hospital, 5021 Bergen, Norway; 10 Department of Medicine, Haraldsplass Deaconess Hospital, 5009 Bergen, Norway; 11 K.G. Jebsen Thrombosis Research and Expertise Center, University of Tromsø, 9019 Tromsø, Norway

**Keywords:** *P. falciparum*, Malaria, T-cell, Neutrophil, Monocyte, Macrophage, HIV, Parasitemia, MPO, sCD25

## Abstract

**Background:**

The immune response during *P. falciparum* infection is a two-edged sword, involving dysregulation of the inflammatory responses with several types of immune cells participating. Here we examined T-cell, monocyte/macrophage and neutrophil activation during *P. falciparum* infection by using soluble activation markers for these leukocyte subsets.

**Methods:**

In a prospective cross-sectional study clinical data and blood samples were collected from adults in Mozambique with *P. falciparum* infection, with (*n* = 70) and without (*n* = 61) co-infection with HIV-1, as well as HIV-infected patients with similar symptoms but without malaria (*n* = 58) and healthy controls (*n* = 52). Soluble (s)CD25, sCD14, sCD163 and myeloperoxidase (MPO) as markers for T-cell, monocyte/macrophage and neutrophil activation, respectively as well as CX3CL1, granzyme B and TIM-3 as markers of T-cell subsets and T-cell exhaustion, were analyzed.

**Results:**

All patient groups had raised levels of activation markers compared with healthy controls. Levels of sCD25 and MPO increased gradually from patient with HIV only to patient with malaria only, with the highest levels in the HIV/malaria group. In the malaria group as a whole, MPO, sCD14 and in particular sCD25 were correlated with disease severity. sCD163, sCD25 and in particular MPO correlated with the degree of parasitemia as assessed by qPCR. Patients with falciparum malaria also had signs of T-cell subset activation (i.e. increased granzyme B and CX3CL1) and T-cell exhaustion as assessed by high levels of TIM-3 particularly in patients co-infected with HIV.

**Conclusion:**

Our data support a marked immune activation in falciparum malaria involving all major leukocyte subsets with particular enhanced activation of neutrophils and T-cells in patients co-infected with HIV. Our findings also support a link between immune activation and immune exhaustion during falciparum malaria, particularly in relation to T-cell responses in patients co-infected with HIV.

**Electronic supplementary material:**

The online version of this article (10.1186/s12879-018-3593-8) contains supplementary material, which is available to authorized users.

## Background

Infection with *Plasmodium falciparum* (*P. falciparum*) is associated with a marked increase in systemic inflammation. Whereas this immune activation could contribute to parasite clearance, an enhanced immune response could also contribute to disease progression by promoting tissue damage rather than protection and through induction of immune exhaustion [1]. The immune response during *P. falciparum* infection involves several immune cells as well as their interactions. Thus, *P. falciparum* infection is associated with a profound T-cell activation with the Th1-derived cytokine interferon γ as a major pathogenic factor [2, 3]. Monocytes/macrophages are one of the main sources of cytokines in malaria-infected individuals and whereas some of these may be of importance for parasite clearance (e.g., interleukin 12) [4], others may be major players in disease progression (e.g., tumor necrosis factor) [5]. Moreover, whereas the inflammatory M1 macrophages could contribute to a sustained and harmful inflammation during *P. falciparum* infection, the anti-inflammatory and pro-resolving M2 macrophages may prevent the development of overt disease. Neutrophils are the most abundant leukocyte population in the bloodstream, the primary compartment of *P. falciparum,* and several studies support a role of these cells in *P. falciparum* infection [6–8]. The release of proteases and myeloperoxidase (MPO) could contribute to tissue damage and parasite invasion, whereas the formation of neutrophil extracellular traps (NETs) could contribute to parasite clearance.

There are several studies on immune activation and inflammation during *P. falciparum* infection, and some of these have also examined T-cells, monocytes/macrophages and neutrophils [9–12], but the role of these cells during falciparum malaria are far from clear. In particular, most studies have focused on one of these cell subsets, and fewer have examined the activation pattern of these cells simultaneously. In the present study we aimed to study the activation of T-cells, monocytes/macrophages and neutrophils during infection with *P. falciparum* by using sCD25, sCD14, sCD163 and MPO as markers of T-cell, monocyte/macrophage and neutrophil activation, respectively. Moreover, whereas sCD163 at least to some degree could reflect M2 activity [13], sCD14 is regarded as a more overall marker of monocytes/macrophage activation. Levels of these markers were related to the degree of parasitemia as assessed by quantitative PCR measurements as well as disease severity. The study was performed in Mozambique that has one of the highest global incidences of co-infection with HIV and falciparum malaria. Co-infection with those two infections poses an increased risk of severe malaria disease [14]. We therefore also examined how HIV infection influenced plasma levels of sCD25, sCD14, sCD163 and MPO in these patients.

## Materials and methods

### Description of study design and participants

The study design has previously been described [14] and the clinical characteristics of the patient groups are given in Table 1. Briefly, in this prospective, cross-sectional study, we included 212 patients admitted into the Medical Emergency Department in the Central Hospital of Maputo, Mozambique during seven months in two malaria peak seasons, from 2011 to 2012. Inclusion criteria were age ≥ 18 years, non-pregnancy, axillary temperature ≥ 38 °C and/or clinical suspected or confirmed malaria infection, and consent from patient or next of kin. Of the 212 screened patients, 131 had *P. falciparum* malaria as assessed by qualitative PCR (*n* = 129) or antigen test/slides (*n* = 2), giving a total of 131 malaria patients. Two patients had in addition double-infection with *P. vivax* and with *P. malariae,* respectively. In the malaria patients, the median age was 37 years (18–84 years), 47% were women, and 53% were co-infected with HIV-1 as assessed by PCR and/or serological tests. For comparison we also included 58 HIV-1-infected patients with suspected, but excluded, malaria. Fifty-two apparently healthy HIV seronegative and malaria negative volunteers with median age 26 years (18–56 years), 41% women, were recruited from hospital employees.

**Table 1:** Clinical characteristics^a)^ of the patient population^b)^

N	HIV only	Malaria only	Malaria & HIV
	58	61	70
Age, years	39 (22–84)	40 (18–79)	40 (20–65)
Sex, females	50 (29/58)	41 (25/61)	50 (35/70)
Hemoglobin (g/dL)	8.9 (2.9–15.2)	11.2 (3.2–17.0)	9.4 (2.5–15.7)
Leukocytes (× 10^9^/L)	8.2 (0.3–25.4)	6.9 (1.3–15.5)	7.8 (0.9–21.8)
Platelets (×10^9^/L)	220 (13–682)	124 (11–452)	90 (8–330)
Se-Creatinine (μmol/L)	161 (41–873)	127 (57–357)	223 (62–1529)
Se-Glucosis (mmol/L)	6.1 (3.3–10.6)	8.7 (3.6–40.5)	6.12 (1.5–27.0)
Liverfailure^c)^	5 (4/57)	5 (3/61)	19 (13/70)
Coagulation disturb.^d)^	0	2 (1/61)	13 (9/70)
Cerebral disturb. ^e)^	33 (19/58)	25 (15/61)	31 (22/70)
Systolic blood pressure	115 (90–160)	122 (70–240)	115 (80–170)
Respiratory rate	29 (12–56)	22 (12–68)	24 (16–42)
Case fatality rate	27.8 (15/54)	1.7 (1/59)	13.0^f)^ (9/69)

^a)^ Values in mean (min-max) or percentage and proportion

^b)^ The 52 healthy controls are not included

^c)^ Defined as jaundice/ bilirubine> 43 μmol/L

^d)^ Defined as bleeding disturbances/ hemolysis

^e)^ Defined as GCS ≤ 11, convulsions or confusion

^f)^ One patient died of non-malarial cause, he is excluded

Of the patients 65% [85/131] had severe malaria and 13% [17/131] had very severe malaria, defined as the presence of ≥1 and ≥ 3 malaria severity criteria, respectively, modified from the World Health Organization [15]. Of the malaria patients 7.6% died (10/128, missing data on outcome in 3 patients).

### Collection of blood samples and preparation of plasma and serum from patients and controls

Blood samples from patients and healthy controls were collected from a pre-alcohol-cleaned peripheral vein into pyrogenic-free tubes with or without EDTA. Plasma tubes were placed immediately on ice, and centrifuged within 30 min at 2000 *g* for 20 min to obtain plasma. Serum samples were allowed to clot for 30 min at room temperature before centrifugation for 10 min at 1000 *g*. Plasma and serum was aliquoted and stored at − 20 °C for 24 h; then at -80 °C. Samples were thawed only once.

#### The qualitative *P. falciparum* PCR in whole blood

Qualitative malaria PCR was performed from the blood cell fraction with the use of malaria plasmodium mitochondria- and species specific 18S PCR, and divergent results were resolved by DNA sequencing [16].

#### The quantitative *P. falciparum* PCR in plasma

The concentration of *P. falciparum* DNA in plasma was measured by quantitative real-time PCR (qPCR) as described [17] using Primer Pf-1 (5′-ATT GCT TTT GAG AGG TTT TGT TAC TTT-3′), primer Pf-2 (5’-GCT GTA GTA TTC AAA CAC AAT GAA CTC AA-3′) and probe Pf (5’-CAT AAC AGA CGG GTA GTC AT-3′) (Applied Biosystems, Cheshire, UK). All samples were run on LightCycler® 480 Multiwell Plate 384, white (Roche Diagnostics, Mannheim, Germany), and sealed with LightCycler® 480 Sealing Foil (Roche). Each run included a positive control and multiple no-template controls. DNA extracted from an external reference material *P. falciparum* (US 03 F Benin I), containing exclusively ring stage parasites in a concentration of 2000 p/μl, was used for dilution series, five-fold, to prepare standard curve for estimating efficiency of the PCR and for quantification.

#### Measurements of cytokines and markers of inflammation

Plasma levels of MPO, sCD14, sCD163, granzyme B, CX3CL1 (fractalkine) and TIM-3 (T cell immunoglobulin and mucin domain 3) and serum levels of sCD25 were measured by enzyme linked immunosorbent assay (ELISA) in duplicate using commercially available antibodies (R&D Systems, Minneapolis, MN, USA) in a 384 format using the combination of a SELMA (Jena, Germany) pipetting robot and a BioTek (Winooski, VT, USA) dispenser/washer (EL406). Absorption was read at 450 nm with wavelength correction set to 540 nm using an ELISA plate reader (Synergy H1 Hybrid, Biotek, Vinooski, VT, USA). The intra- and interassay coefficient of variation were < 10% for all assays.

### Statistical analyses

The distribution of each leukocyte marker was skewed and nonparametric statistics were used throughout. For comparison between the diagnostic groups, Kruskal Wallis was used a priori followed by Dunn’s multiple comparison test between individual groups. Wilcoxon matched-pairs signed rank test was used to compare changes from baseline to follow-up within each diagnostic group. Spearman correlation was used to assess associations between variables. A two-sided *p* < 0.05 was considered significant.

## Results

### MPO, sCD25, sCD14 and sCD163 in *P. falciparum* infection with and without HIV infection

**Fig. 1:**
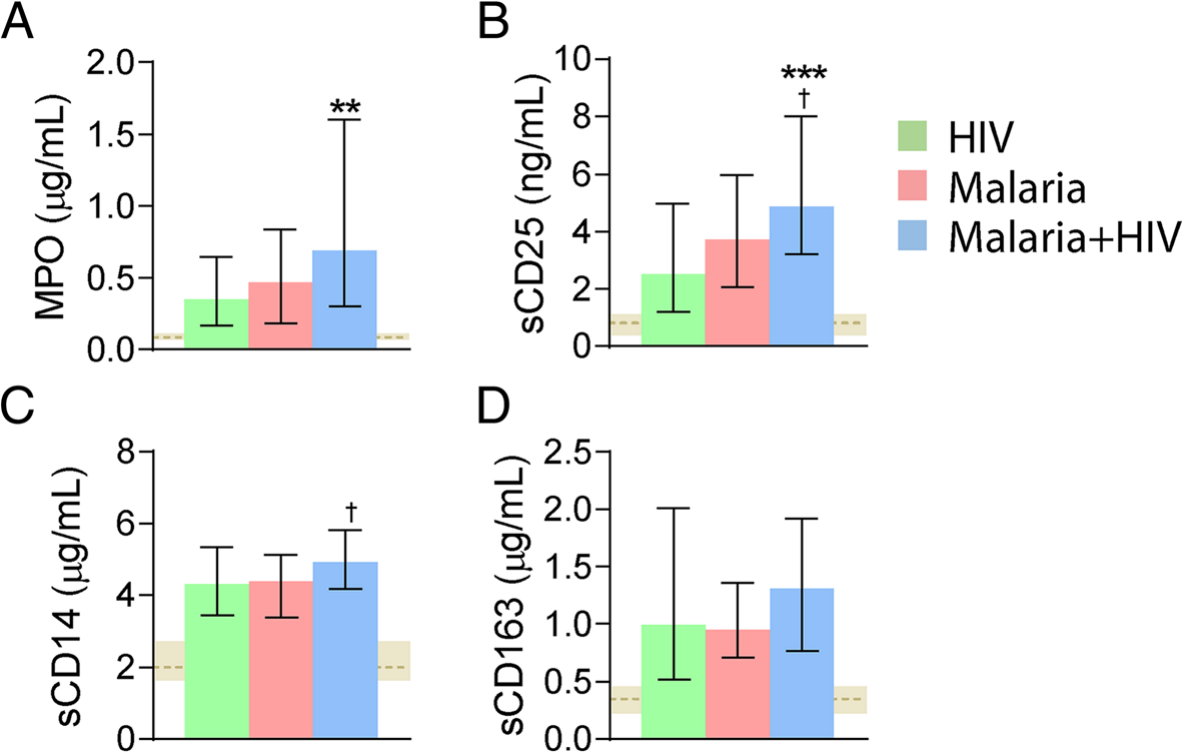
Levels of soluble leukocyte activation markers in patients. Plasma/serum levels of MPO (**a**), sCD25 (**b**), sCD14 (**c**) and sCD163 (**d**) in patients with HIV infection without malaria (*n* = 58), patients with falciparum malaria without (*n* = 61) and with HIV infection (*n* = 70). Data are given as median and 25-75th percentiles. **p < 0.01 and ***p < 0.001 versus HIV without malaria. ^†^*p* < 0.05 versus falciparum malaria without HIV. The horizontal grey shaded area represent levels 25-75th percentiles in age- and sex-matched healthy controls (*n* = 52). In all three groups of patients, all parameters were significantly raised compared with levels in controls (*p* < 0.001 for all comparisons)

As can be seen in Fig. 1, all soluble markers of leukocyte activation were markedly increased in all groups of patients (HIV without malaria [*n* = 58], and malaria with [*n* = 70] and without [*n* = 61] HIV) as compared with healthy controls (*n* = 52). However, some differences were revealed between the different markers. First, sCD25, reflecting T-cell activation, and MPO, reflecting neutrophil activation, showed a gradual increase from the group with isolated HIV infection to the group with malaria and with the highest levels in the group with HIV and malaria co-infection. Second, as for the monocyte markers sCD14 and sCD163, the differences between the three patient groups were modest with the difference in sCD14 between malaria with and without HIV infection as the only significant finding (Fig. 1).

### Soluble markers of leukocyte activation during follow-up

**Fig. 2:**
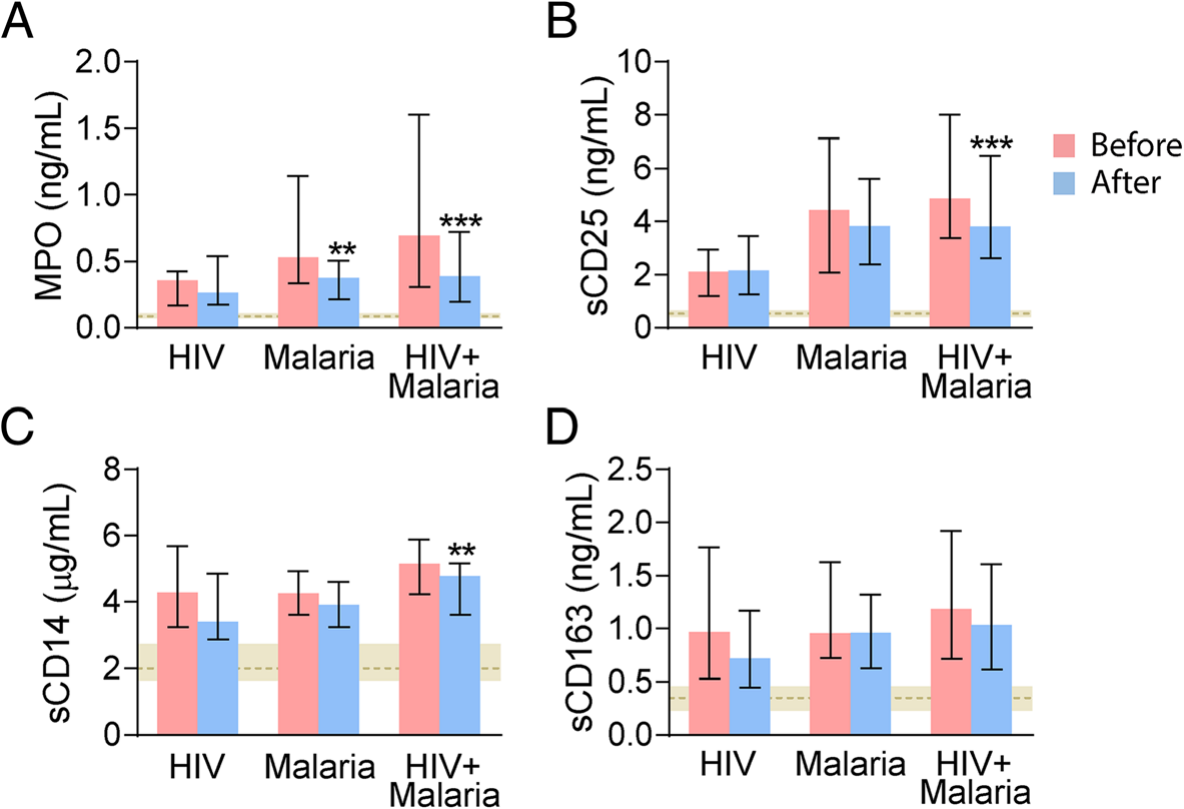
Levels of soluble leukocyte activation markers at admission and follow-up. Plasma/serum levels of MPO (**a**), sCD25 (**b**), sCD14 (**c**) and sCD163 (**d**) in patients with HIV infection without malaria (*n* = 49), patients with falciparum malaria without (n = 6) and with HIV infection (*n* = 22) at admission (before) and 48 h thereafter (after). Data are given as median and 25-75th percentiles. **p < 0.01 and ***p < 0.001 versus levels at admission. The horizontal grey shaded area represent levels 25-75th percentiles in age- and sex-matched healthy controls (n = 52). In all three groups of patients, all parameters were significantly raised compared with levels in controls both at admission and during follow-up (*p* < 0.001 for all comparisons)

In 77 patients (HIV without malaria [*n* = 49], malaria only [n = 6], malaria and HIV [*n* = 22]) we also had follow-up samples taken in hospital after 48 h (Fig. 2). All four markers of leukocyte activation were markedly increased during follow-up in all three groups of patients as compared to healthy controls, despite some decline during follow-up. In HIV-infected patients co-infected with *P. falciparum* there was a significant decline in MPO, sCD25 and sCD14 levels during follow-up, and for MPO, this was also seen for malaria patients without HIV infection. No changes in these markers were seen during follow-up for HIV-infected patients without malaria. None of the three patient groups showed any significant changes for sCD163.

### MPO, sCD25, sCD14 and sCD163 in relation to clinical disease severity

In the malaria group as a whole (*n* = 131), both MPO (*r* = 0.19, *p* = 0.03), sCD14 (*r* = 0.24, *p* = 0.006) and in particular sCD25 (*r* = 0.29, *p* = 0.001), but not sCD163 (*r* = 0.15, *p* = 0.1), were correlated with disease severity as assessed by the WHO definition [15], with the strongest correlation for sCD14 (*r* = 0.33, *p* = 0.011) in those without HIV infection (*n* = 61), and for sCD25 (*r* = 0.27, *p* = 0.022) in those with HIV co-infection (*n* = 70) (Table 2).

**Table 2:** Correlation between disease severity and leukocyte activation markers in patients with (n = 70) and without (n = 61) HIV

	Malaria	Malaria + HIV
sCD14	0.33^a^*(0,011)*	0.08 *(0,533)*
sCD25	0.23 *(0,079)*	0.27^a^ *(0,022)*
MPO	0.13 *(0,316)*	0.21 *(0,078)*
sCD163	0.05 *(0,704)*	0.17 *(0,175)*

^a^Correlation is significant at the 0.05 level (2-tailed)

### MPO, sCD25, sCD14 and sCD163 in relation to the degree of malaria parasitemia

**Fig. 3:**
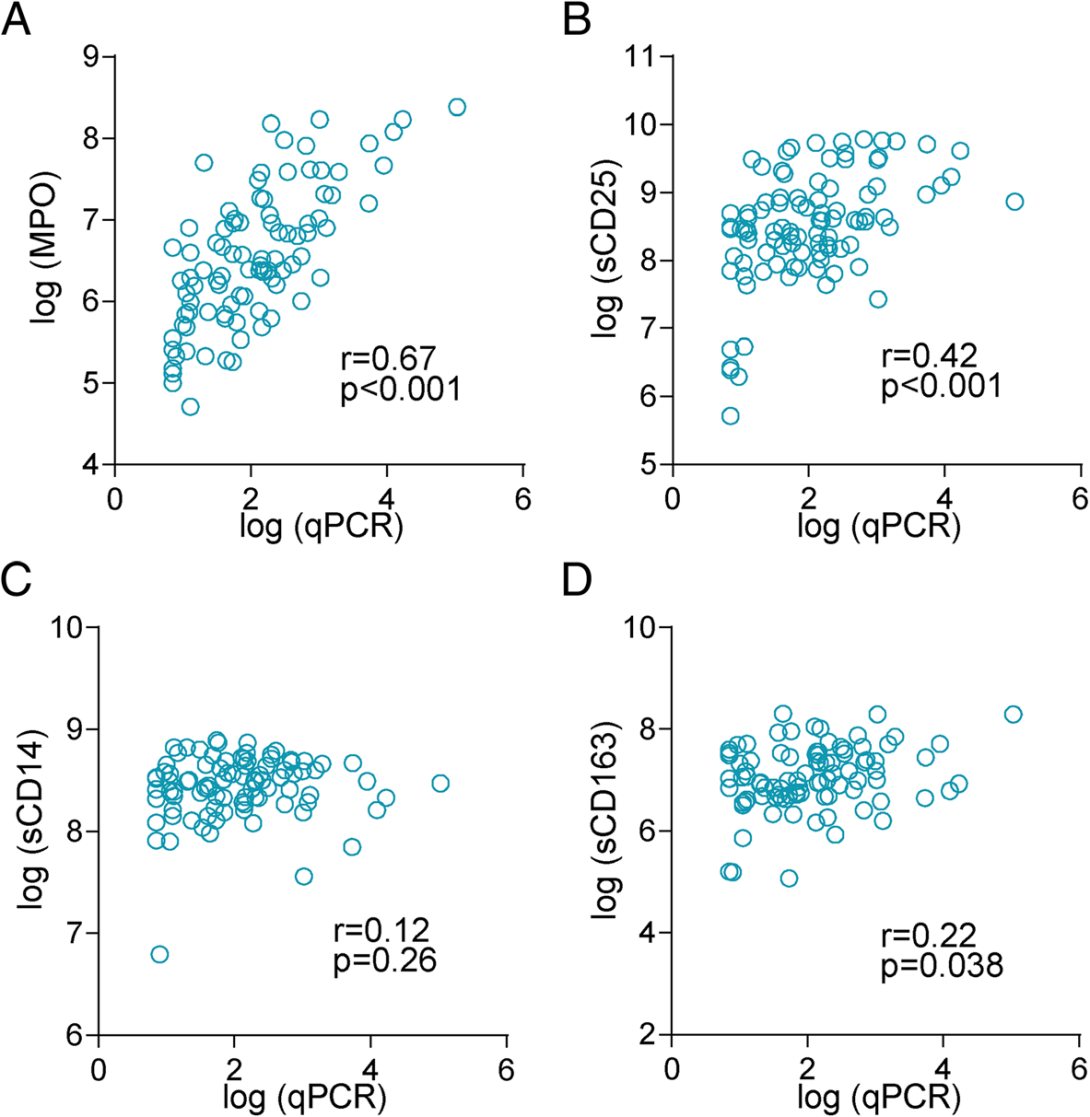
Correlations between soluble leukocyte activation markers and degree of parasitemia. Correlations between MPO **(a**), sCD25 (**b**), sCD14 (**c**) and sCD163 (**d)** and the degree of parasitemia could be assessed by quantitative *P. falciparum* PCR in 93 of the malaria patients (39 patients had levels below the detection limit of the assay). The figure present data from these 93 patients (49 with and 44 without co-infection with HIV)

In 93 of the malaria patients, the degree of malaria parasitemia could be assessed by qPCR (39 patients had levels below the detection limit of the assay). As shown in Fig. 3, sCD163, sCD25 and in particular MPO levels, but not sCD14 levels, were strongly correlated with the degree of parasitemia. For sCD25 and MPO, the same patterns were seen when those with (*n* = 49) and without (*n* = 44) co-infection with HIV were analysed separately (Additional file 1: Table S1).

### Markers of T-cell subset activation and T-cell exhaustion during falciparum malaria

**Fig. 4:**
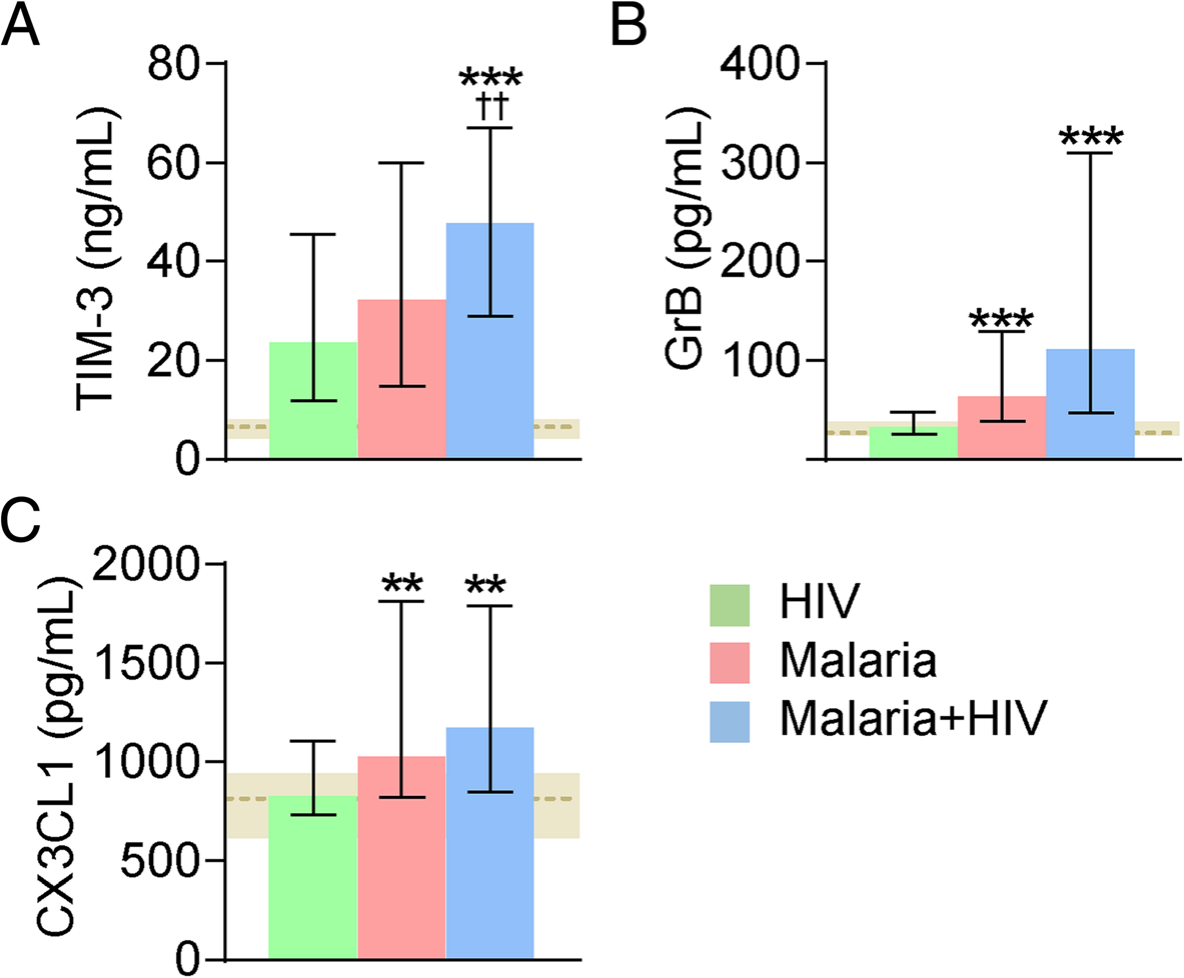
Markers of T-cell subsets activation and T-cell exhaustion during falciparum malaria. Plasma levels of TIM-3 (**a**), granzyme B (GrB) (**b**) and fractalkine (CX3CL1) (**c**) in patients with HIV infection without malaria (n = 58), patients with falciparum malaria without (n = 61) and with (n = 70) HIV infection. Data are given as median and 25-75th percentiles. **p < 0.01 and ***p < 0.001 versus HIV without malaria. ^††^*p* < 0.01 versus falciparum malaria without HIV. The horizontal grey shaded area represent levels 25-75th percentiles in age- and sex-matched healthy controls (n = 52). For the malaria with and without HIV infection, all markers were raised compared with levels in controls (*p* < 0.001). For the HIV group without malaria, TIM-3 but not GrB or CX3CL1, was raised compared to controls (*p* < 0.001)

In order to further characterize the T-cell response during falciparum malaria we measured granzyme B as a marker of activated CD8^+^ T-cells [18] and TIM-3 that could promote induction of effector memory T-cells [19]. Moreover, CX3CL1 could potentially promote differentiation into T effector cells (Teff), a cell type that has high expression of CX3CR1 [20], and could therefore at least indirectly be related to these T-cell subsets. T-cell exhaustion is an important feature of severe malaria [1] and granzyme B [21] and in particular TIM-3 [22] could also be markers of this process. As seen in Fig. 4, malaria patients had increased plasma levels of granzyme B and CX3CL1 as compared with HIV-infected patients without malaria with no difference between those with and without co-infection with HIV. Moreover, TIM-3 levels were also markedly increased during falciparum malaria as compared with HIV-infected patients without malaria, and notably, malaria patients co-infected with HIV had particular high levels of TIM-3 (Fig. 4).

## Discussion

Infection with *P. falciparum* is characterized by a marked immune activation that may be both beneficial (i.e., anti-parasitic) and harmful (i.e., tissue destruction) for the host. We have previously demonstrated an approximately 30-fold increase in interferon inducing protein (IP-10) levels compared with healthy controls in this population of patients with falciparum malaria [23] indicating a marked immune response affecting T-cell activation in these patients. Herein we extend these findings by showing enhanced activation of neutrophils, T-cells and monocyte/macrophages as assessed by the soluble activation markers MPO, sCD25, sCD14 and sCD163, with particular high levels of MPO and sCD25 in malaria patients that were co-infected with HIV. MPO, sCD25 and sCD14 were significantly correlated with clinical disease severity and sCD25 and in particular MPO were also strongly associated with degree of parasitemia as assessed by quantitative *P. falciparum* PCR in plasma. Finally, patients with falciparum malaria also had elevated plasma levels of granzyme B and CX3CL1 suggesting enhanced activation of CD8^+^ T-cells and effector memory T-cell subsets, and the marked increase in TIM-3 suggest some degree of T-cell exhaustion during falciparum malaria particularly in those co-infected with HIV. Our findings further underscore the link between immune activation and immune exhaustion during severe falciparum malaria potentially contributing to disease severity.

MPO and neutrophils have been implicated in the pathogenesis of falciparum malaria. A recent study of *Plasmodium yoelii* non-lethal infection in wild-type and MPO deficient mice as a murine malaria model, suggested that MPO could contribute to a protective anti-parasite response [24]. However, very few studies have analyzed MPO levels in human falciparum malaria. Recently, Rocha et al. showed that circulating neutrophils from malaria patients are highly activated, as indicated by a strong type I interferon transcriptional signature, increased expression of surface activation markers, enhanced release of ROS and MPO, associated with increased liver damage [25]. Herein we show a strong association between MPO levels and the degree of *P. falciparum* parasitemia as assessed by qPCR analyses, suggesting that neutrophils and MPO could promote rather than attenuate parasitemia. Moreover, if the high MPO levels reflect degranulated and exhausted neutrophils, the outcome could also be harmful to the host. MPO release has more recently been related to NET formation that could have anti-parasitic effects, but NETs could also promote vascular pathology during falciparum malaria, illustrating the immune response as a double edge sword during falciparum malaria [26].

We have previously shown that co-infection with HIV during falciparum malaria is associated with enhanced inflammation, increased malaria severity and death [14, 23]. Several other studies also demonstrate that falciparum malaria is more severe in HIV co-infected patients, in particular in those with decreased CD4 T-cell counts [27, 28]. Here we extend these findings by showing that HIV-infected patients with falciparum malaria have markedly raised levels of MPO and sCD25 as compared with both malaria patients without HIV infection and HIV-infected patients without malaria. Moreover, sCD25 levels were also significantly correlated with disease severity in particular in those co-infected with HIV. These findings suggest that despite T-cell deficiency, co-infected patients have signs of sustained T-cell activation. In fact, sustained T-cell activation with increased spontaneous release of inflammatory markers could itself contribute to immunodeficiency. Thus, high circulating levels of sCD25 are mostly accompanied by decreased membrane expression of CD25 on T-cells [29] resulting in attenuated proliferation and IL-2 release upon further challenge by additional stimuli in the microenvironment in vivo such as during falciparum malaria. Also, we found markedly increased TIM-3 levels during falciparum malaria as compared with HIV-infected patients without malaria, with the highest levels in those co-infected with HIV, suggesting some degree of T-cell exhaustion during falciparum malaria in particular in those co-infected with HIV. CD8^+^ T-cell-mediated responses seem to be of major importance in the anti-parasitic T-cell responses and our findings of enhanced granzyme B levels could reflect an anti-malaria effect in these patients. Indeed, very recently granzyme B was found to contribute to the cytotoxic CD8^+^ T-cell-mediated killing of *P. vivax*-infected reticulocytes [30]. However, granzyme B has also been implicated in the development of cerebral malaria [31] and the increased granzyme B levels could also reflect degranulated and exhausted CD8^+^ T-cells, further illustrating the dual effects of the immune response during malaria infection.

Recent studies have demonstrated that monocytes/macrophages are involved in both protection and immunopathology during malaria infection. Data on sCD14 and sCD163 in human falciparum malaria are, however, relatively scarce. As for sCD163 most data are in children and in pregnant women showing some association with disease severity and birth weight in offspring of pregnant women [32]. Herein, all three groups (HIV infection only, falciparum malaria only, combined HIV/malaria infection) had elevated sCD14 and sCD163 levels with only minor differences between the groups. As for sCD14, however, we found that patients with falciparum malaria and HIV had higher levels than those with malaria alone, and sCD14 was also associated with clinical disease severity in the malaria group as a whole. Moreover, we found a significant association between sCD163 and the degree of parasitemia. The interpretation of these later data is difficult and could be by chance, but it could also be speculated that an increased anti-inflammatory M2 response could enhance parasitemia. Our data suggest the involvement of monocyte/macrophage activation during human *P. falciparum* malaria, but the modest changes and the lack of differences in sCD14 between febrile HIV-infected patients and malaria patients suggests that these data should be interpreted with caution.

The present study has some limitations such as potential selection bias of not including the poorest and the wealthiest patients, lack of CD4 cell count on several HIV-infected patients and even if most patients were severely ill, relatively few died, and the sample sizes are too low to probe associations with mortality. Moreover, correlation analyses do not necessarily mean any causal relationship. Also, soluble markers are only surrogate markers of leukocyte action and may not fully reflect their activation status. In relation to T-cell exhaustion, we should ideally have isolated T-cells from fresh patient samples and investigated responses like loss of IL-2 production, loss of proliferative capacity along with co-expression of inhibitory receptors like PD-1 and CTLA-4 [33] Also, stronger diagnostic tools than measurements of sCD163, such as flow cytometry and functional studies in addition to mRNA analyses of isolated monocytes/macrophages are needed to discriminate between M1 and M2 macrophages.

## Conclusions

Our findings support that falciparum malaria is characterized by a marked immune activation involving all major leukocyte subsets, i.e. T-cells, monocytes/macrophages and neutrophils, with particular enhanced activation of neutrophils and T-cells in patients that were co-infected with HIV. Our findings also support a link between immune activation and immune exhaustion during falciparum malaria, particular in relation to T-cell responses in patients co-infected with HIV. Future research in this area should more precisely examine these issues and such clinical studies need to include analyses of leukocyte cell subsets isolated from patients with falciparum malaria in addition to serum and plasma samples.

## Additional file

Additional file 1: **Table S1.** Correlation between degree of parasitemia and leukocyte activation markers in malaria patients alone (*n* = 44) and in malaria patients co-infected with HIV (*n* = 49). (DOCX 13 kb)

## Abbreviations

CX3CL1: fractalkine
MPO: myeloperoxidase
NETs: neutrophil extracellular traps
*P. falciparum*: *Plasmodium falciparum*
qPCR: quantitative real-time PCR
ROS: reactive oxygen species
Teff: T effector cells
TIM-3: T cell immunoglobulin and mucin domain 3

## References

[1] Wykes MN, Horne-Debets JM, Leow CY, Karunarathne DS. Malaria drives T cells to exhaustion. Front Microbiol 2014;27(5):249. PMID: 24904561 10.3389/fmicb.2014.00249 PMC4034037 24904561

[2] Hunt NH, Grau GE. Cytokines: accelerators and brakes in the pathogenesis of cerebral malaria. Trends Immunol 2003;24(9):491–499. Review. PMID: 12967673 10.1016/s1471-4906(03)00229-1 12967673

[3] Belnoue E, Potter SM, Rosa DS, Mauduit M, Grüner AC, Kayibanda M, et al. Control of pathogenic CD8+ T cell migration to the brain by IFN-gamma during experimental cerebral malaria. Parasite Immunol 2008;30(10):544–553. PMID: 18665903 10.1111/j.1365-3024.2008.01053.x 18665903

[4] Malaguarnera L, Imbesi RM, Pignatelli S, Simporè J, Malaguarnera M, Musumeci S, et al. Increased levels of interleukin-12 in Plasmodium falciparum malaria: correlation with the severity of disease. Parasite Immunol 2002;24(7):387–389. PMID: 12164825 10.1046/j.1365-3024.2002.00478.x 12164825

[5] Schofield L, Hackett F. Signal transduction in host cells by a glycosylphosphatidylinositol toxin of malaria parasites. J Exp Med 1993;177(1):145–153. PMID: 8418196 10.1084/jem.177.1.145 PMC2190877 8418196

[6] Brown J, Smalley ME. Inhibition of the in vitro growth of *Plasmodium falciparum* by human polymorphonuclear neutrophil leucocytes. Clin Exp Immunol. 1981;46(1):106–109. PMID: 7039878 PMC1536323 7039878

[7] Greve B, Lehman LG, Lell B, Luckner D, Schmidt-Ott R, Kremsner PG. High oxygen radical production is associated with fast parasite clearance in children with Plasmodium falciparum malaria. J Infect Dis 1999;179(6):1584–1586. PMID: 10228089 10.1086/314780 10228089

[8] Feintuch CM, Saidi A, Seydel K, Chen G, Goldman-Yassen A, Mita-Mendoza NK, et al. Activated neutrophils are associated with pediatric cerebral malaria vasculopathy in Malawian children. MBio 2016 Feb;7(1):e01300–e01315. PMID: 26884431 10.1128/mBio.01300-15 PMC4791846 26884431

[9] Requena P, Barrios D, Robinson LJ, Samol P, Umbers AJ, Wangnapi R, et al. Proinflammatory responses and higher IL-10 production by T cells correlate with protection against malaria during pregnancy and delivery outcomes. J Immunol 2015 Apr 1;194(7):3275–3285. PMID: 25725110 10.4049/jimmunol.1401038 25725110

[10] Dobbs KR, Embury P, Vulule J, Odada PS, Rosa BA, Mitreva M, et al. Monocyte dysregulation and systemic inflammation during pediatric falciparum malaria. JCI Insight. 2017;2(18). pii: 95352. PMID: 28931756 10.1172/jci.insight.95352 PMC5621919 28931756

[11] Tangteerawatana P, Krudsood S, Kanchanakhan N, Troye-Blomberg M, Khusmith S. Low monocyte to neutrophil ratio in peripheral blood associated with disease complication in primary Plasmodium falciparum infection. Southeast Asian J Trop Med Public Health 2014;45(3):517–530. PMID: 24974635 24974635

[12] Erdman LK, Cosio G, Helmers AJ, Gowda DC, Grinstein S, Kain KC. CD36 and TLR interactions in inflammation and phagocytosis: implications for malaria. J Immunol 2009;183(10):6452–6459. PMID: 19864601 10.4049/jimmunol.0901374 PMC2853812 19864601

[13] Ambarus CA, Krausz S, van Eijk M, Hamann J, Radstake TR, Reedquist KA, et al. Systematic validation of specific phenotypic markers for in vitro polarized human macrophages. J Immunol Methods 2012;375(1–2):196–206. PMID: 22075274 10.1016/j.jim.2011.10.013 22075274

[14] Berg A, Patel S, Aukrust P, David C, Gonca M, Berg ES, et al. Increased severity and mortality in adults co-infected with malaria and HIV in Maputo, Mozambique: a prospective cross-sectional study. PLoS One 2014;9(2):e88257. PMID: 24505451 10.1371/journal.pone.0088257 PMC3914956 24505451

[15] WHO. Guidelines for the treatment of malaria. Third ed. 2015:76. http://www.who.int/malaria/publications/atoz/9789241549127/en/.

[16] Haanshuus CG, Mohn SC, Mørch K, Langeland N, Blomberg B, Hanevik K. A novel, single-amplification PCR targeting mitochondrial genome highly sensitive and specific in diagnosing malaria among returned travellers in Bergen, Norway Malar J 2013;12:26. PMID: 23336125 10.1186/1475-2875-12-26 PMC3556099 23336125

[17] Imwong M, Hanchana S, Malleret B, Rénia L, Day NP, Dondorp A, et al. High-throughput ultrasensitive molecular techniques for quantifying low-density malaria parasitemias. J Clin Microbiol 2014;52(9):3303–3309. PMID: 24989601 10.1128/JCM.01057-14 PMC4313154 24989601

[18] Nowacki TM, Kuerten S, Zhang W, Shive CL, Kreher CR, Boehm BO, et al. Granzyme B production distinguishes recently activated CD8(+) memory cells from resting memory cells. Cell Immunol 2007;247(1):36–48. PMID: 17825804 10.1016/j.cellimm.2007.07.004 PMC2134935 17825804

[19] Sabins NC, Chornoguz O, Leander K, Kaplan F, Carter R, Kinder M, et al. TIM-3 engagement promotes effector memory T cell differentiation of human antigen-specific CD8 T cells by activating mTORC1. J Immunol 2017;199(12):4091–4102. PMID: 29127145 10.4049/jimmunol.1701030 PMC5713500 29127145

[20] Gerlach C, Moseman EA, Loughhead SM, Alvarez D, Zwijnenburg AJ, Waanders L, et al. The chemokine receptor CX3CR1 defines three antigen-experienced CD8 T cell subsets with distinct roles in immune surveillance and homeostasis. Immunity 2016;45(6):1270–1284. PMID: 27939671 10.1016/j.immuni.2016.10.018 PMC5177508 27939671

[21] Djoba Siawaya JF, Chegou NN, van den Heuvel MM, Diacon AH, Beyers N, Van Helden et al. differential cytokine/chemokines and KL-6 profiles in patients with different forms of tuberculosis. Cytokine 2009;47(2):132–136. PMID: 19570688 10.1016/j.cyto.2009.05.016 19570688

[22] Yi JS, Cox MA, Zajac AJ. T-cell exhaustion: characteristics, causes and conversion. Immunology 2010;129(4):474–481. PMID: 20201977 10.1111/j.1365-2567.2010.03255.x PMC2842494 20201977

[23] Berg A, Patel S, Gonca M, David C, Otterdal K, Ueland T, et al. Cytokine network in adults with falciparum malaria and HIV-1: increased IL-8 and IP-10 levels are associated with disease severity. PLoS One 2014;9(12):e114480. PMID: 25503583 10.1371/journal.pone.0114480 PMC4263737 25503583

[24] Theeß W, Sellau J, Steeg C, Klinke A, Baldus S, Cramer JP, et al. Myeloperoxidase Attenuates Pathogen Clearance during *Plasmodium yoelii* Nonlethal Infection. Infect Immun. 2016;85(1). pii: e00475–e00416. PMID: 27795354 10.1128/IAI.00475-16 PMC5203641 27795354

[25] Rocha BC, Marques PE, Leoratti FM, Junqueira C, Pereira DB, Antonelli LR, et al. Type I interferon transcriptional signature in neutrophils and low-density granulocytes are associated with tissue damage in malaria. Cell Rep 2015;13(12):2829–2841. 10.1371/journal.pone.0114480. eCollection 2014. PMID: 25503583 10.1016/j.celrep.2015.11.055 PMC4698035 26711347

[26] Boeltz S, Muñoz LE, Fuchs TA, Herrmann M. Neutrophil extracellular traps open the Pandora's box in severe malaria. Front Immunol 2017;8:874. PMID: 28804484 10.3389/fimmu.2017.00874 PMC5532516 28804484

[27] Chalwe V, Van Geertruyden JP, Mukwamataba D, Menten J, Kamalamba J, Mulenga M, et al. Increased risk for severe malaria in HIV-1-infected adults, Zambia. Emerg Infect Dis. 2009;15(5):749; quiz 858. PMID: 19402961 10.3201/eid1505.081009 PMC2687012 19402961

[28] Cohen C, Karstaedt A, Frean J, Thomas J, Govender N, Prentice E, et al. Increased prevalence of severe malaria in HIV-infected adults in South Africa. Clin Infect Dis 2005;41(11):1631–1637. PMID: 16267737 10.1086/498023 16267737

[29] Hofmann B, Nishanian P, Fahey JL, Esmail I, Jackson AL, Detels R, et al. Serum increases and lymphoid cell surface losses of IL-2 receptor CD25 in HIV infection: distinctive parameters of HIV-induced change. Clin Immunol Immunopathol 1991;61(2 Pt 1):212–224. PMID: 1680589 10.1016/s0090-1229(05)80025-x 1680589

[30] Junqueira C, Barbosa CRR, Costa PAC, Teixeira-Carvalho A, Castro G, Sen Santara S, et al. Cytotoxic CD8+ T cells recognize and kill Plasmodium vivax-infected reticulocytes. Nat Med 2018;24(9):1330–1336. PMID: 30038217 10.1038/s41591-018-0117-4 PMC6129205 30038217

[31] Haque A, Best SE, Unosson K, Amante FH, de Labastida F, Anstey NM, et al. Granzyme B expression by CD8+ T cells is required for the development of experimental cerebral malaria. J Immunol 2011;186(11):6148–6156. PMID: 21525386 10.4049/jimmunol.1003955 21525386

[32] Chua CL, Brown GV, Hamilton JA, Molyneux ME, Rogerson SJ, Boeuf P. Soluble CD163, a product of monocyte/macrophage activation, is inversely associated with haemoglobin levels in placental malaria. PLoS One 2013;8(5):e64127. PMID: 23717548 10.1371/journal.pone.0064127 PMC3661483 23717548

[33] Wherry EJ, Kurachi M. Molecular and cellular insights into T cell exhaustion. Nat Rev Immunol 2015;15(8):486–499. PMID: 26205583 10.1038/nri3862 PMC4889009 26205583

