# Supplementary material for: Soluble markers of neutrophil, T-cell and monocyte activation are associated with disease severity and parasitemia in falciparum malaria
Source: BMC Infect Dis. 2018 Dec 18;18:670. doi: 10.1186/s12879-018-3593-8 (PMC6299500; doi:10.1186/s12879-018-3593-8)
Supplement: Supplementary file 1 — Table S1. Correlation between degree of parasitemia and leukocyte activation markers in malaria patients alone (n = 44) and in malaria patients co-infected with HIV (n = 49). (DOCX 13 kb) [file 12879_2018_3593_MOESM1_ESM.docx]

**Supplemental**

**Table S1.** Correlation between degree of parasitemia and leukocyte activation markers in malaria patients alone (n=44) and in malaria patients co-infected with HIV (n=49).

|  | **Malaria** | **Malaria + HIV** |
| --- | --- | --- |
| **sCD14** | 0,157  *(0,313)* | 0,061 *(0,683)* |
| **sCD25** | 0,301* *(0,047)* | 0,461***(0,001)* |
| **MPO** | 0,591** *(0,000)* | 0,711***(0,000)* |
| **sCD163** | 0,055 *(0,728)* | 0,200 *(0,172)* |

* Correlation is significant at the 0.05 level (2-tailed).

** Correlation is significant at the 0.01 level (2-tailed).
